# Computing paths and cycles in biological interaction graphs

**DOI:** 10.1186/1471-2105-10-181

**Published:** 2009-06-15

**Authors:** Steffen Klamt, Axel von Kamp

**Affiliations:** 1 Max Planck Institute for Dynamics of Complex Technical Systems, D-39106 Magdeburg, Germany

## Abstract

**Background:**

Interaction graphs (signed directed graphs) provide an important qualitative modeling approach for Systems Biology. They enable the analysis of causal relationships in cellular networks and can even be useful for predicting qualitative aspects of systems dynamics. Fundamental issues in the analysis of interaction graphs are the enumeration of paths and cycles (feedback loops) and the calculation of shortest positive/negative paths. These computational problems have been discussed only to a minor extent in the context of Systems Biology and in particular the shortest signed paths problem requires algorithmic developments.

**Results:**

We first review algorithms for the enumeration of paths and cycles and show that these algorithms are superior to a recently proposed enumeration approach based on elementary-modes computation. The main part of this work deals with the computation of shortest positive/negative paths, an NP-complete problem for which only very few algorithms are described in the literature. We propose extensions and several new algorithm variants for computing either exact results or approximations. Benchmarks with various concrete biological networks show that exact results can sometimes be obtained in networks with several hundred nodes. A class of even larger graphs can still be treated exactly by a new algorithm combining exhaustive and simple search strategies. For graphs, where the computation of exact solutions becomes time-consuming or infeasible, we devised an approximative algorithm with polynomial complexity. Strikingly, in realistic networks (where a comparison with exact results was possible) this algorithm delivered results that are very close or equal to the exact values. This phenomenon can probably be attributed to the particular topology of cellular signaling and regulatory networks which contain a relatively low number of negative feedback loops.

**Conclusion:**

The calculation of shortest positive/negative paths and cycles in interaction graphs is an important method for network analysis in Systems Biology. This contribution draws the attention of the community to this important computational problem and provides a number of new algorithms, partially specifically tailored for biological interaction graphs. All algorithms have been implemented in the *CellNetAnalyzer *framework which can be downloaded for academic use at .

## Background

Graphs provide simple but often useful formal representation of biological networks capturing one-to-one relationships between biological entities [1]. Different classes of graphs make it possible to incorporate different levels of knowledge. Protein-protein interaction networks, for example, are usually stored as simple undirected graphs. For studying graph-theoretical properties of metabolic networks (which are per se hypergraphs) one often uses directed (and mostly bipartite) graphs. More quantitative relationships are captured by Bayesian networks, an example for directed weighted graphs.

For networks with signal or information flow such as signal transduction or (gene) regulatory networks, *interaction graphs *are the graph model of choice. Interaction graphs (also termed *influence graphs*) are directed signed graphs where each edge (or arc) carries a + or - sign indicating a directed causal relationship between the two involved players, e.g. "molecule A activates or inhibits another molecule B". Interaction graphs are widely used; they serve often as illustrative maps in databases or textbooks and help to represent and to interrogate qualitative knowledge. Apart from statistical properties [2], important functional network properties can be derived from these models:

a) Feedback loops: they are the sources of complex dynamics [3,4]. Recently, Kwon and Cho [5] showed that coherent coupling of feedback loops might be a design principle of cell signaling networks devised to achieve robustness.

b) Signaling paths show the different positive and negative routes along which a molecule can affect another.

c) Dependency matrix: stores for each ordered pair (A, B) of nodes an attribute summarizing the global (direct and indirect) dependence of B upon A [6]. For example, A is an activator of B if at least one positive path from A to B exists but no negative one.

d) (Minimal) cut sets: for a given set of feedback loops or signaling paths one may compute a set of interventions interrupting the signal flow in them [6].

Even though interaction graphs are qualitative models, they also play an important role in representing and analyzing structural relationships of dynamic models. A key property of systems formally described with ordinary differential equations is the Jacobian matrix **J**(**x**) and its sign structure, sgn(**J**(**x**)), which gives rise to an interaction graph: If **J**_*ik*_(**x**)≠0 then an edge from *k *to *i *is drawn and sgn(**J**_*jk*_(**x**)) gives the sign of the edge [4]. In general, sgn(**J**(**x**)) depends on the state **x **where **J**(**x**) is evaluated, but in many biological examples it is constant for all (positive) **x **rendering it a structural invariant. In Systems Biology, where the description of kinetic rate laws is usually hampered by limited knowledge on kinetic parameters and mechanistic details, such structural invariants provide a great opportunity to derive network properties that are independent on this uncertain information. In fact, some fundamental systems properties on qualitative dynamics can be derived from the underlying interaction graph of **J**(**x**):

e) Multistationarity (the coexistence of at least two steady states in a dynamic system) requires a positive feedback in sgn(**J**(**x**)) [3,4,7].

f) Another multistationarity theorem has been given by Craciun *et al*. [8]. Even though it is based on unsigned graphs, it requires the analysis of certain cycles in the graph (which can be mapped back to positive/negative cycles in the associated interaction graph).

g) Oscillations require a negative feedback of length two or higher in sgn(**J**(**x**)) [4].

h) Systems behave monotone with respect to changes in the initial conditions if no (undirected) negative cycle is contained in the interaction graph of **J**(**x**) [9].

i) The initial and steady state response of a system upon perturbations can partially be derived from the interaction graph [10].

Actually all the listed applications of interaction graphs require either an enumeration of all paths/loops in the network (a, b, d, f) or the determination of the shortest positive and shortest negative paths and cycles (or just the information whether certain paths or cycles exist at all) as in (c, e, g, h, i). Although of fundamental importance, these algorithmic problems have so far been discussed only to a minor extent in Systems Biology. Algorithms for the enumeration of paths and cycles have been developed already in the 70s and we will start with a short review on them. We will also compare these standard algorithms with an alternative approach proposed recently [6]. The main part of this work is devoted to the computation of shortest positive and negative paths and cycles in signed graphs. Whereas algorithms for the determination of shortest paths and cycles in unsigned graphs are well-known and of polynomial complexity, in signed graphs, this problem (where we have to distinguish between positive and negative paths and cycles) is much more complicated and is, in general, NP-complete. There are only very few references dealing with this problem. Here we will introduce new algorithm variants which provide either, in polynomial time, approximations of the real shortest paths and cycles or are improvements for finding the exact solution. Using various examples of biological interaction graphs, we demonstrate the performance of these algorithms and show that even in larger networks the exact solution can be found in reasonable time.

## Results and Discussion

### Definitions

We summarize some standard terminology and notations from graph theory. A *graph G *= (*V*, *E*) consists of a set *V *of nodes (vertices) and a set *E *of edges between those nodes. Here we are only concerned with finite graphs meaning that *V *and *E *are finite. In *undirected graphs*, an edge *e *∈ *E *is a pair of nodes: *e *= {*u*, *v*}; *u*, *v *∈ *V, whereas in directed graph*s (*digraphs*) an edge (or arc) is an *ordered *pair *e *= (*u*, *v*) giving it a direction from one node (*u*) to another (*v*). Edges may have additional properties: In a *weighted graph *every edge has a weight (length) represented by a real number (in general the weight can be zero or negative but is typically positive). In a *signed graph *every edge carries either a + or - sign indicating in biological graphs a causal relationship between both species. It is possible that the same pair of nodes may be connected by a positive and negative edge in parallel. All three edge properties – direction, weight and sign – can be combined independently giving rise to different classes of graphs. Herein we will mainly be concerned with the analysis of signed (unweighted) digraphs (= *interaction graphs*), albeit we will sometimes also refer to methods from the other classes.

A *walk *is an alternating sequence of nodes and edges *v*_0_, *e*_1_, *v*_1_, *e*_2_, ... *e*_*n*_, *v*_*n *_(starting and ending with a node) which fulfills the condition that the nodes *v*_*i*-1 _and *v*_*i *_are connected by the edge *e*_*i *_(with the appropriate direction in a digraph). A *trail *is a walk in which no edge occurs twice. A *path *is a trail in which additionally no node occurs twice. The property that a node must not occur twice is sometimes emphasized by calling a path 'simple' or 'elementary'. Here we use the path/trail distinction which makes the use of additional attributes unnecessary. Finally, a *cycle *is a closed trail with no repeated nodes except for the first and last node which must be identical.

Concrete paths or cycles are here written as alternating sequences of nodes and arrows, e.g. A → B → C, which gives a unique identifier for a path or cycle if no parallel edges exist between the involved nodes.

The *length *of a path/cycle is calculated by summing up the edge weights while its *sign *is obtained by multiplying the edge signs. A signed digraph is therefore not equivalent to a weighted digraph with positive and negative edge weights. We will denote the overall sign of a concrete path or cycle by a superscript sign at the end node, e.g. A → B → C → A^-^.

A *strongly connected component (strong component, SCC) *is a maximal subgraph of a digraph in which a path between every pair of distinct nodes exists. The SCCs of a graph can be computed in linear time with Tarjan's algorithm [11]. In a digraph, every cycle lies in exactly one SCC (either the cycle is itself a SCC because every node can be reached from any other node or it is embedded in a larger SCC). Also, every node belongs to exactly one SCC (a single node can also constitute a SCC, e.g. in a digraph without cycles every node is a SCC).

An important concept in relation to signed graphs is *balance *[12]. A signed undirected graph is called *balanced *when every cycle in the graph is positive. A directed graph is balanced if the underlying undirected graph is balanced and it is *cycle-balanced *when all directed cycles are positive (hence, cycle-balance is weaker than balance). It can be proven that a signed digraph *G *is cycle-balanced if and only if every SCC of *G *is balanced (Theorem 13.11, [12]).

### Algorithms for enumeration of paths and cycles

When we are interested in a full enumeration of paths and cycles we need not to distinguish between unsigned and signed graphs. For the latter, paths and cycles can always be computed in the underlying unsigned graph and the overall sign for each path and cycle can easily (in linear time) be attributed afterwards by counting the negative edges involved in the path or cycle.

All paths starting in a certain (seed) node can be generated by performing a breadth-first or depth-first traversal starting from that node. Although this method is easy to implement, the number of paths in a graph can, depending on its structure, quickly explode which can make exhaustive enumeration impractical.

Specialized algorithms for the enumeration of all cycles in a digraph have been developed by various authors, e.g. Tarjan [13] and Johnson [14]. They typically rely on backtracking strategies and reduce the search space through temporary blocking of nodes. Johnson's algorithm is the more efficient variant and has a time complexity that is proportional to the number of cycles in the graph where the proportionality constant is the number of nodes and edges (i.e. the algorithm is linear in the output size but usually exponential in the input size because the number of cycles can increase exponentially with network size). In particular, Johnson's algorithm successively determines the strongly connected components of the graph and removes previous start nodes so that the next iteration leads to new cycles.

**Figure 1:**
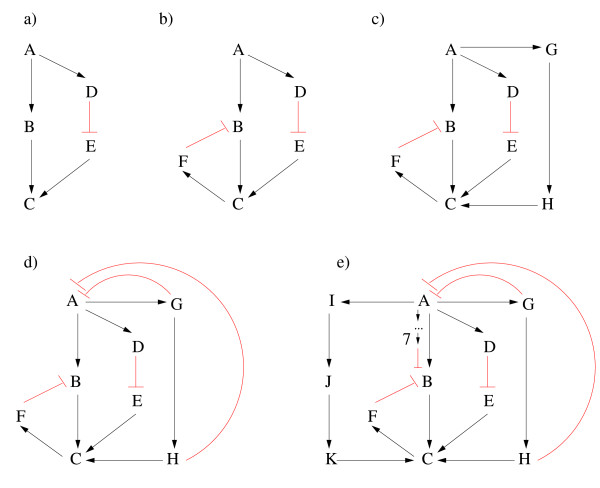
**Example graphs illustrating the different stages and possible problems when searching for shortest paths in signed graphs (for discussion see main text)**. Edges with arrows are positive, those with bars are negative. In Figure 1e, a negative path from A to B with length 7 is indicated.

Since there can be a great number of paths and cycles in a graph and often not all of them are relevant for the question at hand we devised an algorithm that allows one to restrict the paths and cycles to be computed. Nodes and edges that must be passed through are termed obligatory nodes and edges. An obligatory edge can be directly transformed into two obligatory nodes by making its start and end node obligatory. In addition, all other outgoing edges of the start node and all other incoming edges of the end node can be deleted. When enumerating cycles it is now sufficient to process the strongly connected component that contains all obligatory nodes (if obligatory nodes occur in different SCCs then no cycles containing all these nodes exist). Before paths are enumerated, all nodes that can neither reach nor be reached from any of the obligatory nodes are deleted. Reachability is thereby tested by executing an (inexpensive) normal shortest-path algorithm before the enumeration (edge signs are ignored in this case). In addition, reachability can be exploited to reduce the search space when the end nodes of the paths are restricted (e.g. when the paths from A to E in Figure 1a are to be enumerated, it is not necessary to follow the edge from A to B because no path from B to E exists).

In Klamt *et al*. [6] it was shown that path and cycle enumeration can be achieved alternatively by elementary-modes computation, which is a procedure that is often used in the analysis of metabolic networks [15]. Briefly, elementary modes (EMs) can be seen as a formalization of metabolic pathways in which substrates are converted into products while intermediary metabolites are strictly balanced (so-called steady state condition). If irreversible reactions are part of an EM then they can only operate in their specified directions. As third condition, EMs are support-minimal, i.e. if a reaction from an EM is removed the remaining reactions of the mode are not able to assemble a pathway fulfilling the steady-state and reversibility condition. Cycle enumeration can be mapped to elementary-modes computation because (directed) cycles can algebraically be represented in an equivalent way as EMs. One considers vectors **c **(so-called circulations) with |*E*| elements fulfilling a conservation-law (equivalent to the steady-state property of EMs)



(with **N **being the incidence matrix of the graph) and a directionality (positivity) condition (equivalent to the irreversiblity constraints in EMs)



The solution space of this linear equality/inequality system defines a polyhedral cone whose extreme rays (which fulfill a similar minimality condition as EMs) correspond to the cycles of the graph. Since elementary modes correspond also to the extreme rays (of the so-called flux cone associated with a stoichiometric network) we could make use of the elementary-modes algorithm and thereby benefit from recent improvements [15-17]. As shown in [6], elementary-modes computation could also be used for enumerating of paths. In a later section ('Performance') we will briefly investigate whether it pays off to use such a strategy for path/cycle enumeration or whether regular graph-algorithms perform better.

### Algorithms for computing shortest signed paths and cycles

The determination of shortest paths in weighted unsigned digraphs is a well-known problem which can be efficiently solved e.g. with Dijkstra's algorithm [18]. If the graph is unweighted (i.e. all edges have the same weight) then even a simple breadth-first search can be used. Shortest-paths algorithms usually determine the paths that originate from a fixed start node (single-source problem). In order to calculate the shortest paths between all pairs of nodes the single-source problem can be simply iterated over all nodes in the graph. Typically, a shortest path algorithm also calculates the shortest cycle back to the start node. Therefore only the shortest path problem is discussed here.

In general, somewhat surprisingly, even the existence problem for negative or positive paths and cycles (between given start and end nodes) in signed digraphs is NP-complete [19]. Obviously, by neglecting the edge signs we could still compute the shortest paths in the underlying unsigned graph. For a given pair of nodes, we can then easily check whether this path is positive or negative. The difficulty in the general case is to find then the shortest path of the opposite sign. However, it is possible to determine the shortest positive and shortest negative paths (and therefore existence) in polynomial time when either the graph does not contain any negative cycles (*double-label algorithm*; see below) or when the graph is undirected. In the latter case the graph is transformed into an unsigned undirected graph by splitting each edge with a positive sign into two edges with half the weight of the original edge. For this type of graph a polynomial time algorithm has been devised that calculates the shortest paths with an even or odd number of edges [20]. Shortest paths with an even number of edges are then the shortest positive paths and those with an odd number of edges the shortest negative ones.

Since herein we are interested in signed *directed *graphs we cannot use these polynomial algorithms. However, as mentioned above, signed digraphs not containing negative cycles can be treated exactly with the polynomial *double-label algorithm*.

#### Double-label algorithm (DLA)

The double-label algorithm (DLA, [21]) is a modification of Dijkstra's shortest path algorithm. Dijkstra's main procedure determines the shortest paths from a selected start node to the other nodes (single-source problem). This procedure is repeated for every node in the graph when dealing with the all-pairs problem. During its operation, the shortest paths to the other nodes are calculated whereby the path lengths of the shortest paths successively increase. The algorithm keeps track of the currently known shortest distances from the start to every other node as well as (optionally) backward pointers that can be used to reconstruct the actual path.

In a signed graph it is necessary to store in each node both the length of the shortest positive (L^+^) and of the shortest negative (L^-^) path together with the associated backward pointers. This is the main feature of the DLA (for pseudo-code see Additional file 1 section1). During the elongation step, it is now necessary to combine the current shortest positive and/or negative path with all positive and negative edges to test which combination yields a shortest positive or negative path to a neighboring node. Assume we want to compute the shortest positive and negative paths from A to all other nodes in Figure 1a. Since we consider an unweighted graph the DLA can run as a breadth-first search. After the first iteration it delivers A → B^+ ^(L^+^(A, B) = 1) and A → D^+ ^(L^+^(A, D) = 1), after the second iteration A → B → C^+ ^(L^+^(A, C) = 2) and A → D → E^- ^(L^-^(A, E) = 2), and after the third iteration A → D → E → C^-^(L^-^(A, C) = 3). The latter path is of length three and thus longer than the path leading from A to C via B, however, the DLA keeps for each node the length of the shortest positive and shortest negative path separately ("double label") and for C we thus finally have: L^+^(A, C) = 2 (with backward pointer to B) and L^-^(A, C) = 3 (with backward pointer to E).

The DLA delivers exact results in polynomial time if the signed digraph does not contain negative cycles (as in Figure 1a). It usually fails if negative cycles are present as illustrated by the graph in Figure 1b (an extension of Figure 1a): Assume we are interested in the shortest paths leading from A to B. During the 4-th iteration, the standard DLA runs into a negative trail from A to B via A → B → C → F → B^- ^containing the negative cycle B → C → F → B^- ^thus visiting B twice. In its simplest form, the DLA would report a negative path from A to B of length 4 which is apparently wrong.

In fact, in the most general case, what the simple DLA (correctly) computes are the shortest positive/negative walks where nodes and even edges may be visited twice (e.g. when searching for the shortest negative path from G to F in Figure 1c). Only if no negative cycle is present in the graph these walks coincide automatically with the shortest positive and shortest negative paths. This raises the question why negative cycles are problematic when determining shortest paths. First of all, consider an acyclic digraph: Assume that the shortest positive and negative paths from A to B and from B to C are known (possibly only one sign variant for each of the two paths exists). With this information the shortest positive or negative path from A to C *via *B can directly be constructed (possibly both sign variants). This circumstance is exploited by the shortest path algorithms and makes it unnecessary to search through all possible paths for the shortest one. Positive cycles do not pose a problem because they would only elongate (with the same sign) an existing shortest path and therefore do not prevent the identification of the shortest path even if the search algorithm does not explicitly employ checks to avoid cycles. In contrast, a negative cycle, can transform a path from a node X to a node Y into a walk from X to Y (with some nodes/edges visited twice) having the opposite sign. Simple DLA does not employ checks for cycles and such a walk could thus wrongly be reported as a shortest path if the real shortest path is longer or, even worse, if no such path exists. However, this can only happen at all for a given pair of nodes if the graph contains a negative cycle and if both a positive and a negative edge sequence between them is reported by the DLA. In this case the shorter of the two is certainly a correctly identified path while the longer sequence could be a walk with repeated nodes/edges. The determined length of the latter can therefore only serve as a lower bound (which implies that no path exists if this bound is ∞). A small modification by which a shortest path can often correctly be found even when a shorter walk exists is described in the following section.

Note that a different problem is posed by *negative edge weights *in *unsigned *weighted digraphs: If a cycle is present whose *sum *of edge weights is negative then a shortest path algorithm is in danger of repeatedly traversing such a cycle thereby shortening the path length indefinitely. Special shortest paths algorithms can detect such situations (e.g. Bellman-Ford). However, negative cycles characterized by a negative sum of edge weights should not be confused with negative cycles considered here which arise by *multiplication *of edge signs.

#### Double-label algorithm with check for cycles (DLACC)

During each elongation step in the double-label algorithm the backward pointers can be used to check whether the current edge sequence closes a cycle and is thus a trail that can be discarded (in simple DLA, this trail might potentially be extended to a walk). This strategy has been employed by Klamt *et al*. [22]. Applied to Figure 1b, the cycle in the trail A → B → C → F → B^- ^would be detected and a negative path from A to B would thus not be reported by the DLA. The modifications needed to extend single-source DLA to DLACC are explained in more detail in Additional file 1 (sections 1 and 2).

DLA with cycle check (DLACC) is still polynomial in time, however, even a check for cycles cannot avoid that the DLA may fail to detect the correct shortest paths. This is illustrated in Figure 1c: In this expanded version of Figure 1b, a negative path from A to B exists, namely A → G → H → C → F → B^-^, but it would not be identified for the following reason: when looking at the shortest paths to/from intermediate node C these are A → B → C^+ ^and C → F → B^- ^which together would include a cycle (B → C → F → B^-^). Therefore, DLACC would correctly discard this trail. Yet, when looking at intermediate node G or H it becomes clear that the correct shortest negative path form A to B could be composed by adding the shortest positive path from A to G or H and the shortest negative path from there to B.

To summarize, for a given pair of nodes, the DLACC can miss existing paths or deliver longer path lengths than the real shortest if the following three conditions are fulfilled: (i) the graph has a negative cycle, (ii) positive and negative paths between both nodes exist, and (iii) there is a segment on a real shortest path that itself is not shortest, i.e. if a shortest signed path from A to B can be written as A → ... → X → ... → Y → ... → B so that the segment X → ... → Y is not a shortest path (with the respective sign) from X to Y.

#### DLACC with transitive inference (DLACC-TI)

The DLACC extension described in the following delivers correct results (still in polynomial time) also for Figure 1c. Note that for this extension the single-source DLACC must have been applied to every node in the graph. First of all, if the graph contains a negative cycle it is ensured that the DLACC will identify at least one negative cycle, namely one with shortest length in the whole network. As stated above, for a given pair of nodes, the DLACC may have missed existing paths or may have delivered longer path lengths if positive *and *negative paths between the start and the end node exist. The unsigned shortest path length will always correctly be identified during the DLACC (it is attributed to either the shortest positive or shortest negative path). Therefore, for all those pairs (A, B) of nodes between which at least a positive path (with length L^+^) or a negative path (with length L^-^) has been found with DLACC we check for the longer path length max(L^-^, L^+^) (allowing also ∞ for one of the two) whether shorter paths can be constructed by concatenating shortest paths that run via any of the other nodes between A and B. This means that positive/negative shortest paths candidates are constructed by concatenating the appropriate positive/negative shortest paths from A to X and X to B where X can be any node except A or B (complexity for the whole network is still polynomial: O(|*V*|^3^)). If such a candidate – identified by transitive relationships – does not contain a cycle and is shorter than the previous shortest path, then the candidate replaces the previous one. The pseudo-code for this transitive inference is given in Additional file 1 (section 3). In Figure 1c, the negative path from A to B via G, H, C and F would now be identified: DLA with cycle check delivers L^+^(A, B) = 1 and L^-^(A, B) = ∞. We would therefore search for a smaller L^-^(A, B). We see that L^+^(A, G) = 1 and L^-^(G, B) = 4. Hence, there might be a path with L^-^(A, B) = 5. Using the backward pointers we have to check that the concatenated path does not involve a cycle (as is the case here) and then we have confirmed that L^-^(A, B) = 5.

Unfortunately, the result of the DLACC with transitive inference in postprocessing (DLACC-TI) is, in general, still only an *approximation *of the true values as can be illustrated with the further extended graphs in Figures 1d and 1e. In Figure 1d the shortest negative paths from G or H to B would run via A (as stored in the backward pointers). Therefore, the shortest negative path from A to B can not be composed by concatenating shortest paths to/from any other nodes (because they would all contain a cycle) resulting in L^-^(A, B) = ∞ instead of 5. In Figure 1e, a negative path from A to B with length 7 exists which will be returned by the DLACC as the current shortest path. Then, a negative path from A to B via I, J, K will be found by the transitive inference. As the latter path is shorter (6) it replaces the one found by DLACC. However, the real shortest path is even shorter (length 5), which means that the length of the found path is only an upper bound for the length of the real shortest path – and this holds for all values found by transitive inference. Again, negative cycles are the cause that we can only give upper bounds (and sometimes even miss the existence of a positive or negative path). However, in realistic biological networks, it turns out that the results of the DLACC-TI are often close (or even equal) to the exact values (see below).

To summarize, the DLACC-TI is an approximative approach with polynomial complexity. It combines the output of the DLACC with a search for transitive relationships that can lead to the identification of paths missed during DLACC. The length it returns for each pair of nodes is exact for the minimum of L^- ^and L^+^, and an upper bound for the other. The latter could be combined with the lower bounds that can be found with simple DLA: If both bounds are finite and coincide then the DLACC-TI has found a shortest path.

#### Exhaustive search and existence of negative cycles

Hansen [21] describes a branch-and-bound strategy that can be used to augment the DLA to identify walks for which a new search has to be conducted to find the real shortest paths. However, in a first naïve implementation this strategy turns out to be very inefficient. In order to get the exact shortest paths length we apply an exhaustive traversal working in a depth-first manner and storing for each node the current shortest positive and negative distance to the start node. It is easy to implement, requires only a linear amount of memory and turns out to be still sufficiently fast for many of the networks that we have analyzed (see below). Pseudo-code for a single-source implementation (which can easily be extended to the all-pairs-problem) is given in Additional file 1 (section 4).

However, exhaustive search may sometimes be impracticable because of combinatorial explosion of paths to be visited. As mentioned above, calculating shortest positive and negative paths in digraphs is only hard when negative cycles are present, i.e. if at least one SCC of the graph is not balanced (cf. Definitions). Whether this is the case is easy to decide by testing every SCC with a simple linear-time algorithm for balance [23]. Briefly, this algorithm employs a breadth-first search which determines whether or not between some pair of nodes two paths with different signs exist. If such paths can be found it can be concluded that a negative cycle exists and that the SCC is unbalanced (otherwise it is balanced). Alternatively, as mentioned above, the DLACC reports automatically whether a negative cycle exists in the network or not (but its complexity is polynomial, not linear).

#### Two-step algorithm (TSA): exact computation of path lengths combining exhaustive and simple search

**Figure 2:**
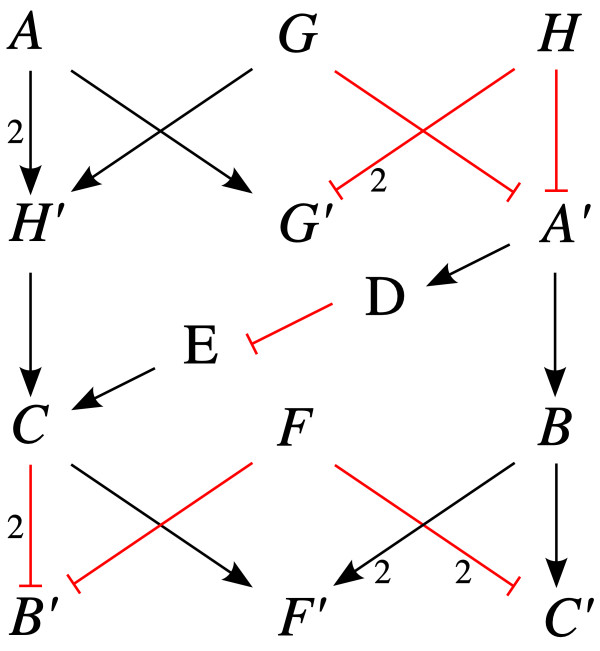
**The graph of Figure 1d transformed for the calculation of shortest paths with the two-step algorithm (TSA)**. The positive edges *X *→ *X' *(*X *∈ {A, B, C, G, H, F}) with zero weight are not displayed to reduce clutter (cf. Additional file 2). The shortest negative path from A to B is *A *→ *H' *→ *C *→ *B' *with a length of 5. Note that the lengths of the (shortest) cycles is computed during the exhaustive search and not displayed in this transformed (acyclic) graph.

The considerations above suggest a method to improve the exact calculation of shortest paths and cycles in signed digraphs (described in the following with respect to the all-pairs problem). First of all, the unbalanced SCCs in the graph are determined (by definition a SCC that consists of a single node is viewed as balanced). Then, separately for each unbalanced SCC, the shortest paths and cycles between the nodes of the SCC are calculated with an exhaustive search (e.g. depth-first traversal as mentioned above). With this information, the nodes and edges of the unbalanced SCCs are then replaced in the following manner (cf. Figures 1d and 2 and Additional file 2): First of all, each node X is split into two variants *X *and *X'*. All incoming edges to X from outside the SCC are connected to *X *whereas the edges going out of the SCC from X now start from *X'*. The node *X *is then connected with all other nodes *Y' *of the SCC with edges that carry the weight of the shortest positive and/or negative paths between the nodes X and Y. In addition, a positive edge *X *→ *X' *with weight zero is added for every pair of split nodes. The resulting transformed graph is free of negative cycles and e.g. the DLA (here applied on a signed digraph with positive weights) can now be used to calculate the remaining shortest positive and negative paths (check for negative cycles is not necessary). A split node is handled in the following way when reading the results: If the path starts at a split node then the *X *variant is chosen and if it ends at such a node the *X' *variant is selected. This *two-step algorithm *(TSA) relies on the fact that a path can pass through any given SCC only once. The reason is that if a path would run through the same SCC twice then the subpath between the two intersections with the SCC would have to be part of this SCC (contradiction). A summary of this procedure is given in Additional File 1 (section 5).

The strategy outlined above shows its largest effect if the network has several smaller SCCs that are separated by regions without cycles. One particular favorable situation is the following: Suppose there are two unbalanced SCCs in the graph and the nodes of the second SCC are reachable from the first SCC (and not vice versa; this is in any case impossible because otherwise the two SCCs would be one giant SCC). If an exhaustive search is applied to the whole graph, then the two SCCs act like a large one because for every path that runs through the first SCC all possible continuations through the second SCC need to be followed. With the strategy above, both SCCs can be independently preprocessed, which in this case can significantly decrease the number of paths that need to be explored.

### Implementation

The described algorithms for cycle and path enumeration and for shortest path computation in signed graphs have been implemented within the framework of *CellNetAnalyzer *(CNA, [22]), a MATLAB toolbox with graphical user interface for network analysis in Systems Biology (free download for academic use via ). CNA calls these algorithms within several routines and they are also available via CNA's application programming interface.

### Performance

Below we discuss benchmark tests of the shortest signed paths and path/cycle enumeration algorithms. Regarding the running times it has to be taken into account that they are implemented with the MATLAB scripting language to make them readily available for *CellNetAnalyzer*. The performance of such scripts is usually significantly lower than when using compiled languages such as C or C++. However, even though the absolute running time should not be considered as state-of-the-art it does allow for a relative comparison of the different algorithms.

We used various test networks for evaluating the performance:

(1) T-cell: interaction graph of a recently published logical model for T-cell receptor signaling [24].

(2) EGFR: interaction graph of a model for EGFR/ErbB signaling constructed in our group [25].

(3) T-cell+EGFR: an artificial interaction graph (with biological characteristics) constructed by linking each node of the output layer of the T-cell network to three randomly selected nodes in the input layer of the EGFR network.

(4) Regulon DB 6.2 [26]: This database contains information about transcription factors and their targets in *E. coli*. Only evidence-based regulation rules where factor and target have associated Blattner numbers and a definitive + or - sign are considered here. For the six transcription factors that consist of two subunits each subunit alone is considered to be able to exert the regulation.

(5) Hippocampal CA1 neuron [27]: The interactions in this network represent signaling pathways and cellular machines of this neuron. Only interactions with a definitive + or - sign are considered here.

(6) Cancer signaling network [28]: This network contains genes and their products which have been found to be relevant during cancer development. Only interactions with a definitive + or - sign are considered here.

The number of nodes and edges in the networks are shown in Table 1.

**Table 1:** Benchmarks for path and cycle enumeration

**Network**	**Nodes**	**Edges**	**Enumeration of cycles**			**Enumeration of input-output paths**		
			
			Number of cycles	EMC	Johnson	Number of I/O paths	EMC	Breadth-first
						
				[s]	[s]		[s]	[s]
T-cell	94	138	100	0.1	0.04	8058	0.98	0.27

EGFR	106	230	237	0.15	0.07	384766	131	21

T-cell+EGFR	200	410	337	6.0 ± 0.9	0.14 ± 0.01	n/a	n/a	n/a

Regulon DB	1493	3565	132	194	0.77	44194	4716	38

CA1 neuron	512	1047	n/a	n/a	n/a	n/a	n/a	n/a

Cancer signaling	1240	3144	n/a	n/a	n/a	n/a	n/a	n/a

The running times when using elementary modes computation (columns "EMC") are compared with those of the graph-algorithms (Johnson's algorithm and breadth-first search, respectively). The number of edges refers to unique edges (parallel edges with the same sign and half-edges are removed before calculation). The values for the combined network "T-cell+EGFR" are mean and standard error over ten runs with different random connections between the two networks. An entry n/a indicates that the procedure quickly ran out of memory (3 GB) because of a combinatorial explosion of paths or cycles. The platform used was MATLAB 2006 b under 32 bit Linux with an Intel E6600 processor.

#### Enumeration of paths and cycles

CNA performs breadth-first traversal for the enumeration of paths. In particular, options to restrict start- and end-nodes or the path length are provided as well as the possibility to calculate only those paths that run via certain nodes and/or edges. For performance measurements the calculation of input-output (I/O) paths is used. These paths connect input nodes (nodes without incoming edge) with output nodes (nodes without outgoing edge). Input and output nodes define the boundaries of the network model. It can be seen in Table 1 that the enumeration of I/O paths is possible – partially in seconds – in medium-scale networks (e.g. EGFR, T-cell). As can also be seen elementary-modes calculation of the paths falls quickly behind breadth-first traversal when the network gets larger (Regulon DB). Note that the number of I/O paths do not simply correlate with the number of nodes and edges in the graph: Although the Regulon DB network has many more nodes and edges than the EGFR network, the latter contains many more I/O paths. In the CA1 neuron, although having fewer nodes and edges than Regulon DB, there are so many I/O paths that full enumeration becomes impractical.

For the enumeration of cycles, CNA now uses Johnson's algorithm. We briefly compare its performance to enumeration via elementary-modes (EM) computation. It can be seen in Table 1 that for cycle enumeration Johnson's algorithm is more efficient, but EM calculation is also practical as long as the number of cycles is not too high. Again, the performance of EM calculation deteriorates quickly for larger networks. More importantly, the running time of Johnson's algorithm is known to scale linearly with the number of cycles whereas the scaling behavior of EM calculation is still an open question.

#### Shortest paths and cycles in signed (interaction) graphs

The following algorithms for computing the shortest positive/negative paths and cycles are implemented in CNA:

• Double-label algorithm with cycle check (DLACC), optionally with transitive inference in postprocessing (DLACC-TI).

• Exhaustive search with depth-first traversal (DFT).

• Two-step algorithm (TSA, mixture of exhaustive search and double-label algorithm).

We compared the performance of these algorithms for the all-pairs problem in the respective test networks (Table 2). Surprisingly, exhaustive search (and thus an exact calculation of all path lengths) is possible in five of the six networks. In the smaller networks (EGFR and T-cell), it requires less than one second and is even faster than DLACC-TI and TSA, since the latter need a certain demand of overhead. This becomes even more significant in the case of Regulon DB. This network contains only 132 cycles which indicates that the network has a rather simple (flat) structure and explains why the exhaustive search is very fast despite the large number of nodes and edges.

**Table 2:** Benchmarks for calculation of shortest signed paths between all pairs of nodes.

**Network**	**Number of uSCCs (with number of nodes)**	**Algorithm**				
		
		approximation with DLACC-TI			TSA	DFT
		
		[s]	TI corrections	remaining errors	[s]	[s]
T-cell	1 (33)	0.79	71	2	0.34	0.02

EGFR	1 (33)	1.18	183	1	0.61	0.26

T-cell+EGFR	2 (33, 33)	6.0 ± 0.04	879 ± 79	3 ± 0	3.1 ± 0.03	419 ± 82

Regulon DB	1 (30)	103	145	0	11.8	1.0

CA1 neuron	1 (154)	25	1869	43*	582*	2213*

Cancer signaling	4 (2, 2, 2, 445)	243	2161	n/a	>12 h	>12 h

The running times for the different algorithms are shown and the quality of the approximation with DLACC-TI is assessed. Also, the number of uSCCS in the networks together with the number of nodes that they contain is shown. In the column "TI corrections" the number of shorter paths that can be identified with transitive inference after having run DLACC is given. The "remaining errors" column shows how many shortest paths (after the TI step) differ in their length compared to the exact results delivered by TSA or DFT. When an algorithm ran longer than 12 hours it was considered impractical and terminated. Therefore, no exact results were determined for the cancer signaling network and consequently the quality of the approximation with DLACC-TI cannot be given (*) For the CA1 neuron, the search depths in the two-step algorithm and in the exhaustive search were limited to length 18 to make calculations practicable. Therefore some paths may have been missed. The longest shortest path identified for this network with the DLACC-TI is also of length 18. The computational environment is the same as in Table 1.

The DLACC-TI algorithm delivering approximative results performs sufficiently well in all networks, in particular in the cancer signaling network where an exact result could not be obtained in reasonable time with exhaustive search or TSA. Only the DLACC-TI can be applied here to get an approximative solution. In general, as can be seen by the number of corrections, transitive inference in postprocessing may strongly reduce the number of incorrect results delivered by the DLACC (especially in the case of the CA1 neuron). Furthermore, the number of remaining errors after DLACC-TI (we can compare the results with those from the exact algorithms except in the cancer signaling network) is very low or even zero (Regulon DB). We conjecture that this is a general property of biological signaling and regulatory networks and is due to the relatively low number of negative feedback loops (compared to what is theoretically possible).

The TSA best exploits situations where at least some SCCs are connected as in the T-cell+EGFR example. T-cell and EGFR alone comprise only one single SCC where TSA cannot lead to a better performance. However, TSA can also be favorable when the search depth is restricted in complicated networks because it is then sufficient to restrict the search depth only when traversing the unbalanced SCCs. This is demonstrated for the CA1 neuron where the two-step algorithm with restricted DFS search achieves the same result as a restricted DFS search of the whole network but uses only one quarter of the computation time.

## Conclusion

The enumeration of paths and cycles (feedback loops) and the calculation of shortest positive/negative paths in interaction graphs are fundamental issues in Systems Biology. Enumeration of paths (breadth-first search) and cycles (Tarjan's and Johnson's algorithm) are standard problems in graph theory. We compared it with enumeration by elementary-modes computation, an algebraic technique borrowed from metabolic network analysis. It turns out that algorithms exploiting explicitly the graph structure (where each edge connects two nodes) are superior to the more general elementary-modes approach which has been developed for hypergraphs where hyperedges (such as the bi-molecular reaction A+B → C+D) may connect more than two nodes.

Apart from full enumeration of paths and cycles, we identified the calculation of shortest positive/negative paths and cycles in (signed) interaction graphs as a key problem for many applications. In contrast to standard shortest path computation, this problem is NP-complete and only very few algorithmic approaches have been described in the literature so far. We proposed here extensions and several new algorithms, for both computing exact results (in smaller and medium-scale networks) and approximations (in large-scale networks). Benchmarks in realistic biological networks showed that exact results can be obtained in networks with up to several hundreds nodes and interactions, a property which one would not expect in random networks. A class of even larger graphs can still be treated exactly by the two-step algorithm combining exhaustive and simple search strategies. Finally, an approximative algorithm (with polynomial complexity) for large networks (where exact solutions cannot be obtained in reasonable time) was introduced herein which seems to deliver results that are very close or even equal to the exact values. Again, this phenomenon can probably be attributed to the particular topology of cellular signaling and regulatory networks which contain only a relatively low number of negative feedback loops.

All algorithms described herein have been implemented in the *CellNetAnalyzer *framework  and are thus publicly available for biological network analysis.

## Authors' contributions

SK initiated this study. Both authors contributed equally in developing, implementing and testing algorithms and in writing the paper. Both authors read and approved the manuscript.

## Supplementary Material

Additional file 1 **Pseudo-code**. Pseudo-codes of shortest paths algorithms in signed directed graphs discussed in the main text. Click here for file

Additional file 2 **Adjacency matrices for transformed graph**. Adjacency matrices (for positive and negative edges) for the transformed graph in Figure 2. Click here for file
