# Supplementary material for: Computing paths and cycles in biological interaction graphs
Source: BMC Bioinformatics. 2009 Jun 15;10:181. doi: 10.1186/1471-2105-10-181 (PMC2708159; doi:10.1186/1471-2105-10-181)
Supplement: Additional file 1 — Pseudo-code. Pseudo-codes of shortest paths algorithms in signed directed graphs discussed in the main text. [file 1471-2105-10-181-S1.pdf]

Supplementary Information

Part 1: Pseudo-Codes

Computing paths and cycles in biological interaction graphs

Steffen Klamt[1§] and Axel von Kamp[1]

[1] Max Planck Institute for Dynamics of Complex Technical Systems, Sandtorstr. 1, D-39106 Magdeburg, Germany
[§]Corresponding author: klamt@mpi-magdeburg.mpg.de

Pseudo-codes of algorithms for computing shortest signed paths

1. Double-label algorithm (DLA)

The nodes are designated with the numbers from 1 to k.

```
function [dist_pos, dist_neg, prev_pos, prev_neg] =
       DLA_single_source(pos_edge_weight, neg_edge_weight, start_node)

  for v from 1 to k // initialization
    dist_pos[v] := infinity
    dist_neg[v] := infinity
    prev_pos[v] := undefined
    prev_neg[v] := undefined
  endfor
  dist_pos[start_node] := 0
  T_pos := the set {1..k}
  T_neg := the set {1..k}
  while T_pos is not empty and T_neg is not empty
    p := node in T_pos with minimal dist_pos[]
    n := node in T_neg with minimal dist_neg[]
    if p <= n // elongate shortest positive path
      remove p from T_pos
      for each neighbor v of p
        if v is element of T_pos
          dist_via_p := dist_pos[p] + pos_edge_weight(p, v)
          if dist_via_p < dist_pos[v]
           (* check for cycle *)
            dist_pos[v] := dist_via_p
            prev_pos[v] := p
          endif
        endif
        if v is element of T_neg
          dist_via_p := dist_pos[p] + neg_edge_weight(p, v)
          if dist_via_p < dist_neg[v]
          (* check for cycle *)
            dist_neg[v] := dist_via_p
            prev_neg[v] := -p
          endif
        endif
      endfor
    else // elongate shortest negative path
      remove n from T_neg
      for each neighbor v of n
        if v is element of T_pos
          dist_via_n := dist_neg[n] + neg_edge_weight(n, v)
          if dist_via_n < dist_pos[v]
           (* check for cycle *)
            dist_pos[v] := dist_via_n
            prev_pos[v] := -n
          endif
        endif
        if v is element of T_neg
          dist_via_n := dist_neg[n] + pos_edge_weight(n, v)
          if dist_via_n < dist_neg[v]
           (* check for cycle *)
            dist_neg[v] := dist_via_n
            prev_neg[v] := n
          endif
        endif
      endfor
    endif
  endwhile
  return dist_pos[], dist_neg[], prev_pos[], prev_neg[]
```

pos_edge_weight[k] and neg_edge_weight[k] may contain non-negative weights different from one; they are infinity if no edge from start_node to k exists.
The (* check for cycle *) marks lines which need to be extended for DLACC (see below). For simplicity, the described algorithm only calculates the shortest paths from start_node to the other nodes v. The shortest positive/negative cycle back to start_node can in addition be obtained with minor modifications.
During execution, dist_pos[v] and dist_neg[v] contain the currently known shortest distances along positive/negative paths from the start_node to v.
prev_pos and prev_neg are backwards pointers. The sign of a backward pointer indicates whether the preceding node is reached via a positive or a negative edge. Backwards pointers can be used for two purposes:

(i) Reconstruction of the actual shortest path after termination.
(ii) Checking for cycles during execution (needed for DLACC, see below).

Both is achieved by tracing back the pointers until the start_node has been reached. As an example, let prev_pos[5]=-3. This means that the current shortest positive path to node 5 comes from node 3. The minus sign indicates that the edge connecting node 3 with node 5 on this path is negative. Therefore, at the next step of the backtracking procedure, the value of prev_neg[3] needs to be looked up.

2. Double-label algorithm with check for cycles (DLACC)

For the DLACC algorithm, a subroutine that checks for cycles on the current path (using `prev_pos` and `prev_neg`) has to be called in the 4 lines marked with `(* check for cycle *)` in the DLA code above. Only if no cycle is found the surrounding **`if`** statement is executed.

3. Double-label algorithm with transitive inference (DLACC-TI)

Execute DLACC for each (start) node and collect the results in the matrices `dist_pos, dist_neg`, `prev_pos` and `prev_neg` (each row contains the results obtained by running DLACC with the corresponding node as start node). Note that `dist_pos` and `dist_neg` may contain errors if the graph has negative cycles. A better approximation can be obtained by transitive inference conducted after the DLACC. The algorithm checks for all triplets of distinct nodes x, y, z whether a shorter pos./neg. path from x to y (compared to those originally stored in `dist_pos` / `dist_neg`) can be found when running from x to z and then from z to y. If a potentially shorter path is found, one has to check (with the help of `pred_pos` and `pred_neg`) that it does not contain a cycle:

```
function [dist_pos, dist_neg] = transitive_inference(dist_pos, dist_neg, pred_pos, pred_neg)

  dist_pos_old := distpos
  dist_neg_old := distneg

  for each node x
    for each node y ≠ x
      for each node z ≠ x,y
        xzydist := dist_pos_old[x,z] + dist_pos_old[z,y]
        if xzydist < dist_pos[x,y]  // potentially shorter pos. path from x to y via z?
          if no cycle_in_augmented path x --> z+ | z+ --> y+  // use prev_pos and prev_neg for cycle check
            dist_pos[x,y] := xzydist;
          endif
        endif
        xzydist := dist_pos_old[x,z] + dist_neg_old[z,y]
        if xzydist < dist_neg[x,y]  // potentially shorter neg. path from x to y via z?
          if no cycle_in_augmented path x --> z+ | z --> y- // use prev_pos and prev_neg for cycle check
            dist_neg[x,y] := xzydist;
          endif
        endif
        xzydist := dist_neg_old[x,z] + dist_pos_old[z,y]
        if xzydist < dist_neg[x,y]  // potentially shorter neg. path from x to y via z?
          if no cycle_in_augmented path x --> z- | z --> y+  // use prev_pos and prev_neg for cycle check
            dist_neg[x,y] := xzydist;
          endif
        endif
        xzydist := dist_neg_old[x,z] + dist_neg_old[z,y]
        if xzydist < dist_pos[x,y]  // potentially shorter pos. path from x to y via z?
          if no cycle_in_augmented path x --> z- | z --> y-  // use prev_pos and prev_neg for cycle check
            dist_pos[x,y] := xzydist;
          endif
        endif
      endfor
    endfor
  endfor
  return dist_pos, distneg
```

4. Computing shortest pos./neg. paths with depth-first traversal

This algorithm computes signed shortest paths from a single start node to the other nodes in the graph (and also the shortest pos./neg. cycles back to the start node) by depth first traversal. Basically, the algorithm checks all possible paths/cycles starting in `start_node`.

```
function [dist_pos, dist_neg] = signed_dfs(pos_edge_weight, neg_edge_weight, start_node)
  for v from 1 to k // initialization
    dist_pos[v] := infinity
    dist_neg[v] := infinity
    cur_neighbor_ind[v] := 1
  endfor

  path := empty stack // stack of triples (node, length, sign); represents the currently active path
  push(path, (start_node, 0, +1));
  while path not empty
    (v, path_length, path_sign) := top(path)
    neighbor_list= neighbors(v); // neighbor_list is array of triples (neighbor, weight, sign)
    if cur_neighbor_ind[v] > length(neighbor_list)  //all neighbors visited
      pop(path)          //remove top entry of the path stack and go back to previous node
      cur_neighbor_ind[v] := 1;
    else
      (next_node, edge_weight, edge_sign) := neighbor_list[cur_neighbor_ind[v]]
      new_length := path_length + edge_weight
      new_sign := path_sign * edge_sign
      cur_neighbor_ind[v] := cur_neighbor_ind[v] + 1
      if next_node == start_node or next_node not yet on path     //new cycle or path found
        if new_sign == +1
          if new_length < dist_pos[next_node]
            dist_pos[next_node] := new_length
          endif
        else // new_sign == −1
          if new_length < dist_neg[next_node]
            dist_neg[next_node] := new_length
          endif
        endif
      if next_node not yet on path     // continue only with paths, not with cycles or trails
        push(path, (next_node, new_length, new_sign))
      endif
    endif
  endwhile
  return dist_pos[], dist_neg[]
```

5. Outline of the two-step algorithm (TSA)

1. Identify the unbalanced strongly connected components (uSCC; see main text).
2. Within each uSCC calculate shortest signed paths between all pairs of nodes using depth-first traversal as given above.
3. Transform graph as described in section 2.3.5 (main text) and illustrated by Supplementary Table 1.
4. Apply DLA (check for cycles not necessary) to the transformed graph.
5. Extract the relevant results.
