# Supplementary material for: Computing paths and cycles in biological interaction graphs
Source: BMC Bioinformatics. 2009 Jun 15;10:181. doi: 10.1186/1471-2105-10-181 (PMC2708159; doi:10.1186/1471-2105-10-181)
Supplement: Additional file 2 — Adjacency matrices for transformed graph. Adjacency matrices (for positive and negative edges) for the transformed graph in Figure 2. [file 1471-2105-10-181-S2.pdf]

Supplementary Information

Part 2: Adjacency matrices of the transformed graph in Figure 2

Computing paths and cycles in biological interaction graphs

Steffen Klamt[1§] and Axel von Kamp[1]

[1] Max Planck Institute for Dynamics of Complex Technical Systems, Sandtorstr. 1, D-39106 Magdeburg, Germany
[§]Corresponding author: klamt@mpi-magdeburg.mpg.de

Adjacency matrices of the transformed graph

Adjacency matrices for positive and negative edges of the transformed graph shown in Figure 2 (obtained from Figure 1d). These matrices serve as input for the double-label algorithm in the second part of the two-step algorithm (after finishing the exhaustive depth-first search). The nodes in the rows are the start-points and those in the columns the end-points of the edges. The values are edge weights; when no value or ∞ is given for a certain node pair this means that the corresponding edge does not exist.

<table>
<tr><th>+</th><th>A</th><th>G</th><th>H</th><th>C</th><th>F</th><th>B</th><th>E</th><th>D</th><th>A′</th><th>G′</th><th>H′</th><th>C′</th><th>F′</th><th>B′</th></tr>
<tr><td>A</td><td rowspan="6" colspan="6">∞</td><td rowspan="6" colspan="2">∞</td><td>0</td><td>1</td><td>2</td><td rowspan="3" colspan="3">∞</td></tr>
<tr><td>G</td><td></td><td>0</td><td>1</td></tr>
<tr><td>H</td><td></td><td></td><td>0</td></tr>
<tr><td>C</td><td rowspan="3" colspan="3">∞</td><td>0</td><td>1</td><td></td></tr>
<tr><td>F</td><td></td><td>0</td><td></td></tr>
<tr><td>B</td><td>1</td><td>2</td><td>0</td></tr>
<tr><td>E</td><td></td><td></td><td></td><td>1</td><td></td><td></td><td></td><td></td><td rowspan="2" colspan="6">∞</td></tr>
<tr><td>D</td><td></td><td></td><td></td><td></td><td></td><td></td><td></td><td></td></tr>
<tr><td>A′</td><td rowspan="3" colspan="3">∞</td><td></td><td></td><td>1</td><td></td><td>1</td><td rowspan="6" colspan="6">∞</td></tr>
<tr><td>G′</td><td></td><td></td><td></td><td></td><td></td></tr>
<tr><td>H′</td><td>1</td><td></td><td></td><td></td><td></td></tr>
<tr><td>C′</td><td></td><td></td><td></td><td rowspan="3" colspan="3">∞</td><td></td><td></td></tr>
<tr><td>F′</td><td></td><td></td><td></td><td></td><td></td></tr>
<tr><td>B′</td><td></td><td></td><td></td><td></td><td></td></tr>
</table>

<table>
<tr><th>−</th><th>A</th><th>G</th><th>H</th><th>C</th><th>F</th><th>B</th><th>E</th><th>D</th><th>A′</th><th>G′</th><th>H′</th><th>C′</th><th>F′</th><th>B′</th></tr>
<tr><td>A</td><td rowspan="6" colspan="6">∞</td><td rowspan="6" colspan="2">∞</td><td>∞</td><td></td><td></td><td rowspan="3" colspan="3">∞</td></tr>
<tr><td>G</td><td>1</td><td>∞</td><td></td></tr>
<tr><td>H</td><td>1</td><td>2</td><td>∞</td></tr>
<tr><td>C</td><td rowspan="3" colspan="3">∞</td><td>∞</td><td></td><td>2</td></tr>
<tr><td>F</td><td>2</td><td>∞</td><td>1</td></tr>
<tr><td>B</td><td></td><td></td><td>∞</td></tr>
<tr><td>E</td><td></td><td></td><td></td><td></td><td></td><td></td><td></td><td></td><td rowspan="2" colspan="6">∞</td></tr>
<tr><td>D</td><td></td><td></td><td></td><td></td><td></td><td></td><td>1</td><td></td></tr>
<tr><td>A′</td><td rowspan="3" colspan="3">∞</td><td></td><td></td><td></td><td></td><td></td><td rowspan="6" colspan="6">∞</td></tr>
<tr><td>G′</td><td></td><td></td><td></td><td></td><td></td></tr>
<tr><td>H′</td><td></td><td></td><td></td><td></td><td></td></tr>
<tr><td>C′</td><td></td><td></td><td></td><td rowspan="3" colspan="3">∞</td><td></td><td></td></tr>
<tr><td>F′</td><td></td><td></td><td></td><td></td><td></td></tr>
<tr><td>B′</td><td></td><td></td><td></td><td></td><td></td></tr>
</table>

Legend: (uSCC = unbalanced strongly connected component; regular nodes are the nodes not belonging to an uSCC and have therefore not been split)

input nodes of uSCCs are not connected among each other

output nodes of uSCCs do not connect to input nodes of the same uSCC

copied from the corresponding parts of the original adjacency matrix

input nodes of uSCCs have no edges pointing to regular nodes

results from the exhaustive depth-first search search in the uSCCs with modified main diagonal to remove the cycles (the zeros in the positive matrix represent the direct $X \rightarrow X'$ ($X \in$ {A, B, C, G, H, F}) edges)

regular nodes have no edges pointing to output nodes of uSCCs

output nodes of uSCCs are not connected among each other

input nodes of an uSCC do not connect to output nodes of another uSCC
